# Comparison of the photocatalytic activity of novel hybrid photocatalysts based on phthalocyanines, subphthalocyanines and porphyrins immobilized onto nanoporous gold†

**DOI:** 10.1039/d1ra01331a

**Published:** 2021-03-18

**Authors:** David Steinebrunner, Günter Schnurpfeil, Jan Thayssen, Jorge Adrian Tapia Burgos, Andre Wichmann, Dieter Wöhrle, Arne Wittstock

**Affiliations:** Institute of Applied and Physical Chemistry, Center for Environmental Research and Sustainable Technology, University Bremen Leobener Str. UFT 28359 Bremen Germany awittstock@uni-bremen.de; MAPEX Center for Materials and Processes, University Bremen Bibliothekstr. 1 28359 Bremen Germany; Organic and Macromolecular Chemistry, University Bremen Leobener Str. NW2 28359 Bremen Germany woehrle@uni-bremen.de; Institute for Organic and Analytical Chemistry, University Bremen Leobener Str. UFT 28359 Bremen Germany

## Abstract

A series of different singlet oxygen photosensitizers was immobilized onto nanoporous gold powder with a mean pore size of 40 nm *via* copper catalyzed azide–alkyne cycloaddition. The attachment of phthalocyanine and porphyrin derivatives was performed on the peripheral substituent of the macrocycle, whereas the subphthalocyanine derivatives were attached *via* the axial substituent with respect to the macrocyclic ring system. All obtained hybrid systems were studied in the photooxidation of 2,5-diphenylfuran as a chemical singlet oxygen quencher and showed increased photocatalytic activity compared to the same amount of the corresponding photosensitizer in solution due to photoinduced interactions of the plasmon resonance of the nanostructured gold support and the attached photosensitizer. The understanding of the different photophysical interactions depending on the coordination mode of the macrocycle as well as the position of the absorbance in the electromagnetic spectrum is an important point in the development towards highly active hybrid photocatalysts covering a broad absorption range within the spectrum of visible light.

## Abstract 2

A set of heterogeneous photocatalysts based on porphyrinoid photosensitizers immobilized onto nanoporous gold was prepared and investigated in respect of differences in photocatalytic singlet oxygen sensitization.

## Graphical abstract

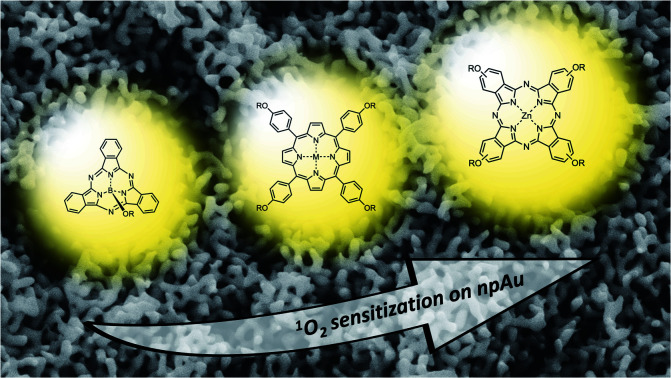

## Introduction

Singlet oxygen (^1^O_2_) as one sort of reactive oxygen species (ROS) is of special interest, for example as a reactant in many modern organic transformations or as active part in photodynamic therapy (PDT).^1–3^ The formation of this ROS is usually achieved either by direct chemical release from ozonides or hydrogen peroxide and sodium hypochlorite or by photochemical sensitization employing various photosensitizers that transfer energy from an excited triplet state to the ground state of oxygen (^3^O_2_, ^3^Σ_g_) under formation of reactive singlet oxygen (^1^O_2_, ^1^Δ_g_).^4^ Apart from all organic photosensitizers like rose bengal or methylene blue, porphyrinoid metal complexes like zinc(ii) phthalocyanine- or zinc(ii) porphyrin derivatives are among the most used and studied photocatalysts for singlet oxygen sensitization.^5,6^ In general, this class of metal complex combines very high extinction coefficients, good triplet state quantum yields and lifetimes with reasonable chemical stability, which is often a problem in the case of the all organic photosensitizers.^5–7^ Since such complexes also suffer from singlet oxygen induced self-decomposition, the lifetime of the photosensitizers can be drastically improved by immobilization on a support, as it was shown in the case of zinc(ii) phthalocyanines when immobilized onto an inert silica support.^8^

A promising new approach towards highly active singlet oxygen sensitizers is the combination of a classical porphyrinoid sensitizer with an optically active support, where the photocatalytic activity can be further improved by interactions between the active support and the immobilized sensitizer.^9,10^ Nanostructured gold systems, as for example gold nanoparticles (AuNPs) or nanoporous gold (npAu), both show strong absorption of visible light due to their surface plasmon resonance and are therefore ideal candidates in the context of such novel hybrid photocatalysts.^9–13^ Whereas colloidal AuNP based hybrid materials behave more like a pseudo homogeneous system, monolithic npAu systems represent a truly heterogeneous material as hybrid photosensitizer. Therefore, AuNP based systems are promising candidates in PDT applications due to their homogeneous behavior and the biocompatibility of AuNPs.^14–16^ npAu on the other hand is a perfect example for a heterogeneous catalyst that can be easily employed in both, batch and flow reactor systems.^17–20^

**Fig. 1:**
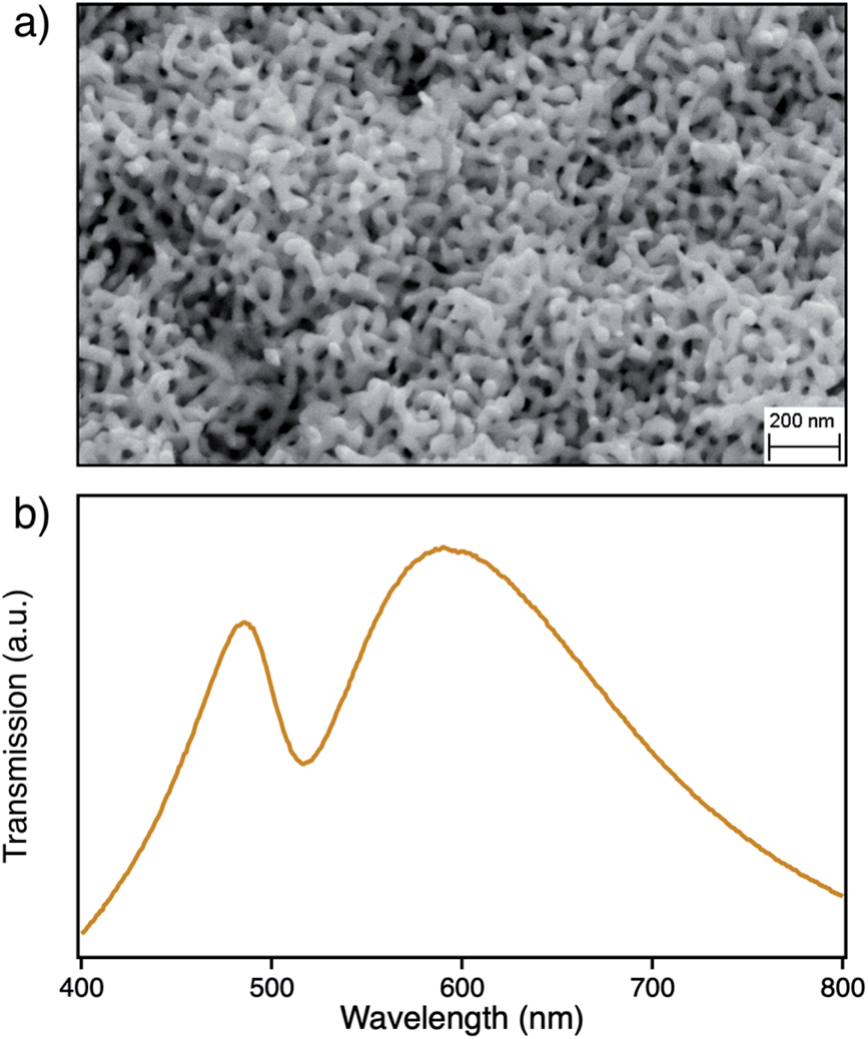
(a) SEM micrograph of the npAu structure with a magnification of 100k; (b) corresponding transmission spectrum of a npAu foil in a DMF environment with same pore sizes showing the longitudinal (490 nm) and transverse (580 nm) absorption of the plasmon resonance.

The nanoporous structure of npAu can be easily obtained by corrosion of a suitable bimetallic alloy such as Ag/Au, where the macroscopic shape of the material is already determined by the shape of the starting alloy.^17–20^ The evolution of nanoporosity is a complex process consisting of leaching of the less noble ad-metal followed by reorganization of the gold atoms in the evolving ligaments.^17–21^ The dealloying time thereby is a crucial parameter for the average size of the obtained pores and ligaments in the material, which also have a direct influence on the position of the surface plasmon resonance.^22–24^ In this study we used a npAu powder with average pore size of around 40 nm (Fig. 1a) showing a typical optical behavior with the longitudinal part of the plasmon resonance at 490 nm and the transverse part at around 580 nm (Fig. 1b).

**Scheme 1:**
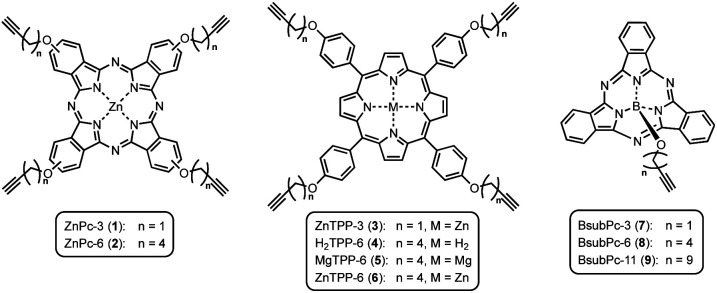
Molecular structures of the different substituted photosensitizers; zinc(ii) phthalocyanines (ZnPcs, 1-2), the tetraphenylporphyrins (TPPs, 3–6) and the boron(iii) subphthalocyanines (BsubPcs, 7–9).

On this active support, different photosensitizers from the classes of phthalocyanines, porphyrins and sub-phthalocyanines (Scheme 1) were immobilized following a two-step approach of self-assembled monolayer (SAM) formation and subsequent attachment *via* copper catalyzed azide–alkyne cycloaddition (CuAAC). The different classes of photosensitizers were selected to obtain various hybrid systems covering the whole range of visible light absorption as a complete set of panchromatic singlet oxygen sensitizing systems.

All of the obtained hybrid systems were studied in the photooxidation of 2,5-diphenylfuran (DPF) as singlet oxygen quencher, allowing a comparison between the different systems. The effect of the position of the sensitizer absorption and the resulting photophysical interactions will be discussed in detail. Besides that, the influence of the alkyl chain length on the photosensitizer and the resulting overall orientation resulting from axial and peripheral sensitizer attachment are of major interest in this study.

## Results and discussion

### Synthesis and characterization

**Scheme 2:**
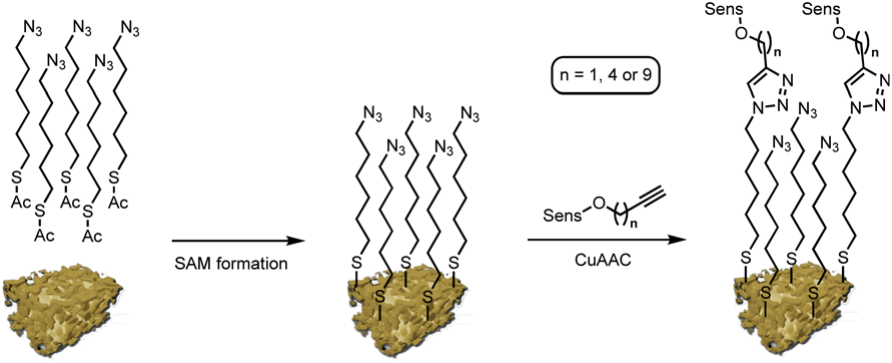
Schematic representation of the npAu hybrid preparation; formation of an azide terminated SAM using 6-azidohexyl thioacetate followed by attachment of the different photosensitizers *via* CuAAC (“click reaction”).

Nanoporous gold powder with a mean pore size of 40 nm was prepared employing a free corrosion method selectively leaching Ag out of an Ag/Au alloy.^9,10^ Organic functionalization of the obtained npAu support was achieved following a two-step approach as reported previously by us (Scheme 2).^9,10^ In a first step, a SAM employing 6-azidohexyl thioacetate as linker unit is prepared on the npAu surface resulting in an azide functionalized surface. In the second step, the different photosensitizers were attached to the azide-terminated SAM *via* CuAAC to give the desired hybrid systems.

All of the obtained hybrid systems were analyzed regarding the pore sizes of the support and the distribution of the immobilized photosensitizer by scanning electron microscopy (SEM) and energy dispersive X-ray (EDX) spectroscopy. The pore sizes were found to stay constant at 40 nm unaffected by the functionalization. The employed photosensitizers all showed a homogeneous distribution over the npAu support, which was shown by EDX mapping and line scan experiments.^10^ For the quantification of the amount of immobilized photosensitizer, the hybrid systems were dissolved in ultra-pure *aqua regia* and subsequently analyzed by inductively coupled plasma mass spectrometry (ICP-MS), determining the fraction of the central metal compared to the support quantity (Table S1†). In the case of immobilized ZnPcs, quantities of around 150 μg g^−1^ hybrid catalyst were found as determined already in earlier studies.^9,10^ For the TPP derivatives slightly lower quantities of around 110 μg g^−1^ catalyst were determined whereas for the BsubPcs quantities of around 500 μg g^−1^ were found due to the higher or lower sterical demand of the macromolecules.

**Scheme 3:**
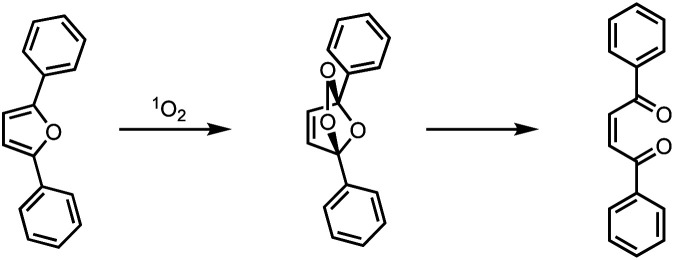
Reaction scheme for the photooxidation of the chemical singlet oxygen quencher 2,5-diphenylfuran (DPF).

The photocatalytic singlet oxygen sensitization activity of the heterogeneous hybrid photocatalysts was determined by irradiation of the catalysts with a 300 W Xe arc lamp and a light intensity of 180 mW cm^−2^ within a self-built setup described elsewhere.^10,25^ For the quantification of ^1^O_2_ formed during the catalyst run, DPF was used as chemical ^1^O_2_ quencher, allowing to follow the reaction progress *via* UV-Vis spectroscopy by the decrease of the DPF absorption band employing Lambert Beer's rule. DPF is structurally related to the standard chemical quencher 1,3-diphenylisobenzofuran (DPBF), reacting selectively with ^1^O_2_*via* an endo-peroxide to a diketo species (Scheme 3). This quencher was used instead of DPBF as the absorption is significantly blue-shifted and therefore irradiation without self-decomposition is also possible below 500 nm, where the main absorption band of the porphyrin derivatives is located. For every DPF photooxidation, the corresponding turnover numbers (TON) and turnover frequencies (TOF) were calculated for quantification of the activity based on the fraction of irradiated photosensitizer as described in detail within the ESI.† Noteworthy, the bare npAu without attached sensitizer showed no catalytic activity, *i.e.*, generation of singlet oxygen under all studied conditions.

### Phthalocyanines on npAu

**Fig. 2:**
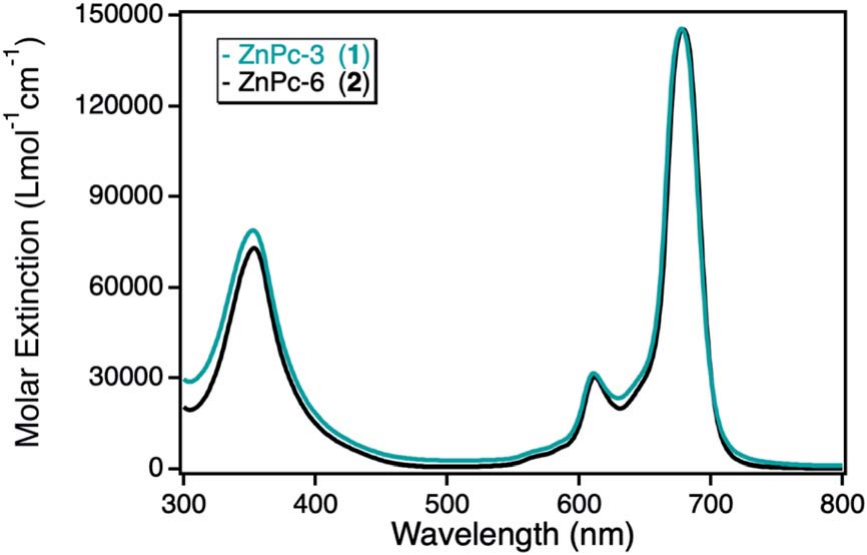
UV-Vis spectra for the photosensitizer zinc(ii) phthalocyanine with either peripheral propynoxy- (1, cyan) or hexynoxy-substituents (2, black) recorded in DMF.

The photocatalytic activity of the zinc(ii) phthalocyanine (ZnPc) derivatives immobilized on various npAu supports had been investigated already in detail in the photooxidation of DPBF.^10,23,25^ However, this class of hybrid material was also analyzed in the photooxidation of DPF, acting as reference system in comparison to the novel hybrid materials employing other macrocyclic metal complexes. For this purpose, two zinc(ii) phthalocyanines containing either propynoxy- (1) or hexynoxy- (2) peripheral substituents were studied in the photooxidation reaction of DPF before and after immobilized on a npAu powder support. The ZnPc derivatives exhibit the typical phthalocyanine absorption behavior with the maximum absorption of the Q-band around 680 nm with high molar extinction coefficients of 140 000 L mol^−1^ cm^−1^ and a weaker absorption at around 360 nm of the Soret-band (Fig. 2).

**Fig. 3:**
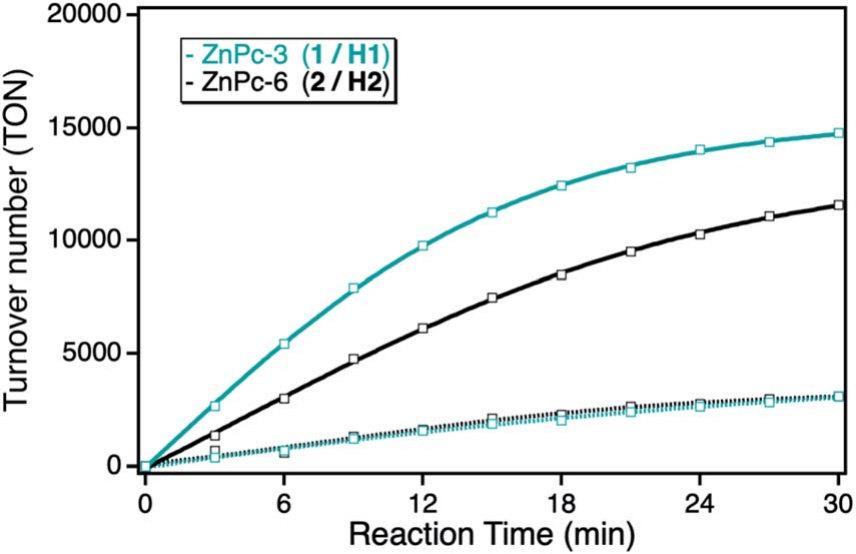
Photocatalytic DPF conversion given as TON *vs.* reaction time employing either the ZnPc with peripheral propynoxy- (1, H1, cyan) or hexynoxy-substituents (2, H2, black) in solution (dashed line) and when immobilized on the npAu support (bold line).

Under panchromatic irradiation between 400 and 800 nm, both ZnPc derivatives show singlet oxygen sensitization responsible for the DPF oxidation. The activity of both derivatives in solution revealed similar TON indicating the same photocatalytic activities, corresponding to a total DPF conversion of around 10% after 30 min irradiation time. In contrast, after immobilization onto the npAu support, both ZnPc hybrid systems showed an increase in activity, even stronger for the hybrid consisting of the propynoxy-substituted ZnPc derivative (Fig. 3). The origin of the increased photocatalytic activity of the ZnPc photosensitizer when immobilized onto the npAu support was shown in earlier studies to be a consequence of the heavy-atom effect as well as energy transfer of the nanostructure caused plasmon resonance to the attached photosensitizer.^10,12,13^

The orientation of the immobilized ZnPc derivatives on the npAu surface was shown to adopt a planar geometry due to the multidentate properties of the peripheral substituents enabling up to four binding sites to the azide groups of the SAM.^25^ Due to the planar orientation, it was also shown that the central zinc ion forms an additional axial coordination to free azide groups of the SAM resulting in a penta-coordinated central ion known for various ZnPc and ZnTPP derivatives.^25–32^ Within this orientation of the immobilized ZnPc, the axial coordination is stronger for the ZnPc derivative with shorter peripheral substituents, explaining the stronger observed increase in photocatalytic activity for the hybrid system H1 in comparison to H2.

### Porphyrins on npAu

For the investigation of different TPP based photosensitizers, zinc(ii) tetraphenylporphyrin (ZnTPP, 3, 6) derivatives with peripheral propynoxy- and hexynoxy-substituents similar to the ZnPc sensitizers were used. In addition, a magnesium(ii) tetraphenylporphyrin (MgTPP, 5) as well as a metal-free tetraphenylporphyrin (H_2_TPP, 4) were chosen to determine the influence of the central metal cation as well as the situation, where the penta-coordinated geometry could not be formed due to the lack of the central metal ion.

**Fig. 4:**
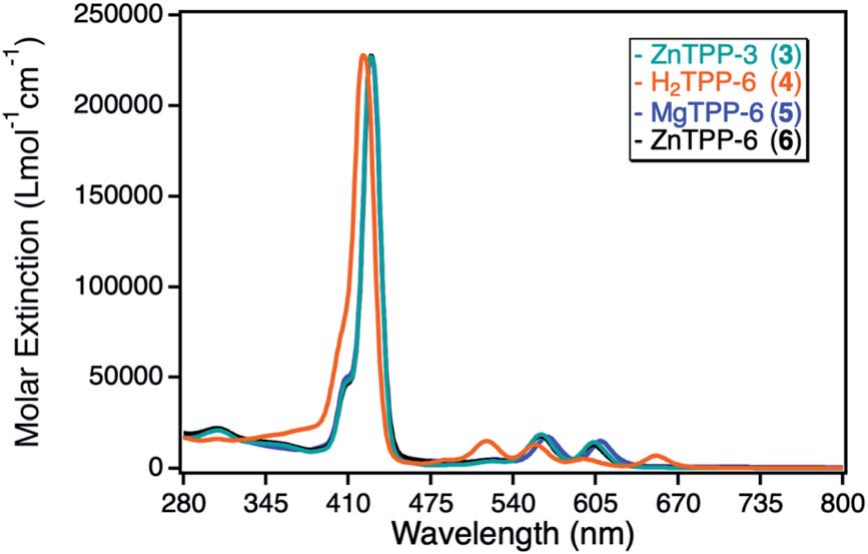
UV-Vis spectra for different tetraphenylporphyrin photosensitizers: zinc(ii) tetraphenylporphyrin with either peripheral propynoxy- (3, cyan) or hexynoxy-substituents (6, black) as well as the magnesium(ii) tetraphenylporphyrin (5, blue) and metal-free tetraphenylporphyrin (4, orange) both bearing peripheral hexynoxy-substituents. All spectra were recorded in DMF.

The UV-Vis spectra of those TPP derivatives all exhibit the typical porphyrinoid absorption characteristics, with the main absorption of the Soret-band around 420 nm and a weaker and splitted Q-band between 500–650 nm. As for the ZnPc derivatives, the ZnTPP sensitizers exhibit identical UV-Vis spectra, with a maximum molar extinction coefficient of 230 000 L mol^−1^ cm^−1^, significantly higher than for the ZnPc sensitizers. The MgTPP exhibits a weak bathochromic shift in respect to the ZnTPP derivatives, whereas the metal-free H_2_TPP shows a hypsochromic shift and an additional splitting of the Q-band, a typical observation for metal-free porphyrinoids.^33^ The molar extinction coefficients for those two derivatives were found in the same range as for the ZnTPP derivatives (Fig. 4).

**Fig. 5:**
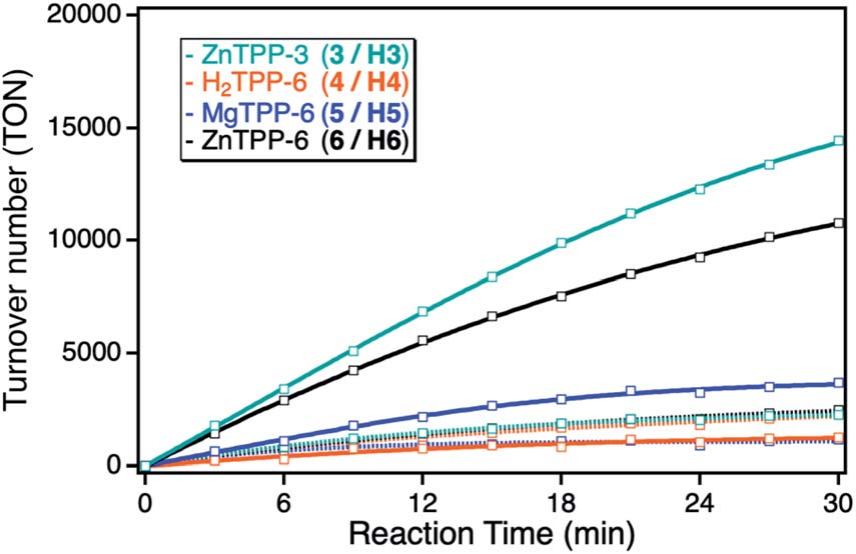
Photocatalytic DPF conversion given as TON *vs.* reaction time employing either the ZnTPP with peripheral propynoxy-(3, H3, cyan), with hexynoxy-substituents (6, H6, black), the MgTPP (5, H5, blue) or the metal-free H_2_TPP (4, H4, orange) in solution (dashed line) and when immobilized on the npAu support (bold line).

Although the ZnTPP sensitizers offer significantly higher extinction coefficients in comparison to the ZnPc sensitizers, the observed photocatalytic activities of the sensitizers in solution showed the same singlet oxygen sensitization activity. This can be explained by the position of the main absorption band at higher energies, leading to a larger energy difference between the excited triplet state of the sensitizer with respect to the singlet oxygen energy level, thus making energy transfer less efficient. After immobilization onto the npAu powder, the ZnTPPs showed a similar behavior as the ZnPcs, again with stronger enhancement for the propynoxy-substituted ZnTPP derivative, but an overall smaller increase in activity in comparison to the ZnPc derivatives (Fig. 5). The weaker increase in activity can be explained by the lower spectral overlap of the ZnTPP absorption band with the plasmon resonance of the npAu support. In addition, the main interaction is expected to proceed *via* the Q-band of the TPP sensitizer, having molar extinction coefficients in the order of one magnitude below the Q-band absorption of the ZnPc derivatives. Nevertheless, the higher activity of the ZnTPP based hybrid H3 in comparison to hybrid H6 indicates a similar penta-coordinated geometry of the central Zn ion as observed for the ZnPc photosensitizers.

The MgTPP derivative showed similar behavior as compared to the ZnTPP derivatives, but with significantly lower activity for both, the sensitizer in solution as well as immobilized on the npAu support. An interesting behavior was observed for the metal-free H_2_TPP derivative, where the direct interaction *via* axial coordination is excluded. In only this case, the observed photocatalytic activity after immobilization onto the npAu was smaller than compared to the photosensitizer in solution. However, the amount of immobilized sensitizer could only be assumed to be the same as for the other TPP derivatives, as the lack of the central metal ion makes the determination *via* ICP-MS impossible.

### Subphthalocyanines on npAu

The class of boron(iii) subphthalocyanines (BsubPcs) used for immobilization was chosen because of their good singlet oxygen sensitization activity and high chemical stability.^34^ The macrocyclic ring system in this class of sensitizers is structurally similar to the common phthalocyanine ring, comprising of only three azomethine-bridged isoindole units around a boron central ion (Scheme 1). In addition, this class of sensitizers offers the possibility of a direct axial linkage to the npAu support, which also results in a non-planar, bowl-shaped geometry of the photosensitizer.^34^

**Fig. 6:**
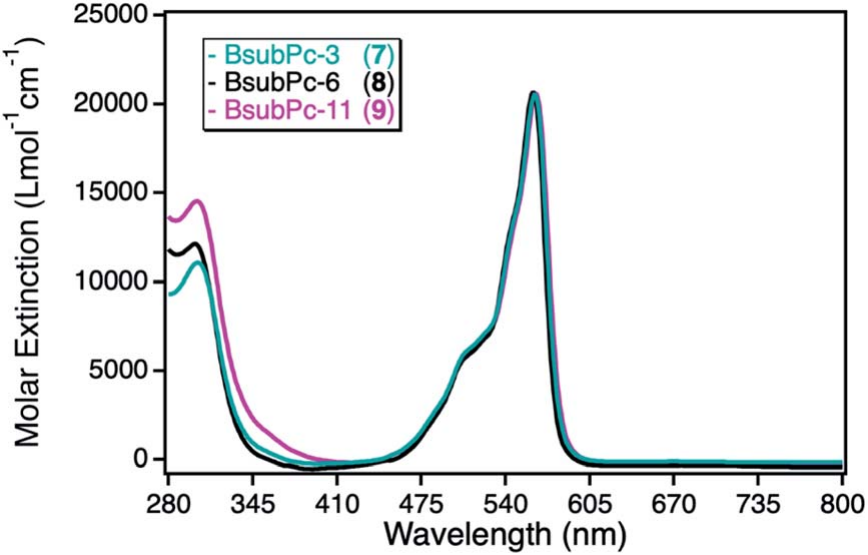
UV-Vis spectra for the photosensitizer boron(iii) subphthalocyanine with either axial propynoxy- (7, cyan), hexynoxy- (8, black) or undecynoxy-substituents (9, magenta) recorded in DMF.

The UV-Vis spectra of the three different BsubPc derivatives 7–9 show similar absorption bands as the ZnPc derivatives, however exhibiting a large blue-shift of both, the Soret- and the Q-band. The strongest absorption band in this class of sensitizer is again the Q-band at around 550 nm (Fig. 6). The molar extinction coefficients are very low in comparison to the parent ZnPc photosensitizers with values around 21 000 L mol^−1^ cm^−1^. On the other hand, those photosensitizers exhibit the largest spectral overlap with the plasmon resonance of the npAu support, making this combination a promising design for a novel hybrid material.

**Fig. 7:**
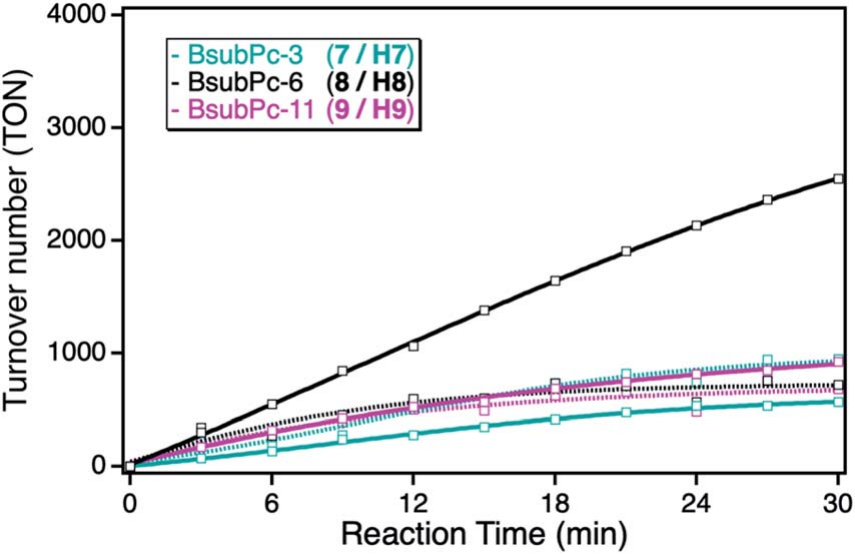
Photocatalytic DPF conversion given as TON *vs.* reaction time employing either the BsubPc with peripheral propynoxy-(7, H7, cyan), with hexynoxy- (8, H8, black) or with undecynoxy-substituents (9, H9, magenta) in solution (dashed line) and when immobilized on the npAu support (bold line).

As consequence of the small extinction coefficients, all of the BsubPc derivatives showed only small singlet oxygen sensitization activities in solution, exhibiting TOF values of around 30 min^−1^. In comparison to the earlier employed ZnPc and ZnTPP photosensitizers, those class of sensitizers showed the lowest overall activity in solution (Fig. 7). However, after immobilization onto the npAu support, a completely different behavior regarding the alkyl chain length of the sensitizer was observed. The reason for this behavior is the different substitution position compared to the phthalocyanine and porphyrin macrocycles. Whereas the binding to the npAu is achieved in peripheral position in the case of the phthalocyanine and porphyrin photosensitizers, in the case of the BsubPcs the binding to the npAu is achieved in axial position. Therefore, the secondary axial coordination point in this case is blocked, leading to the situation where not only the chain length of the SAM determines the distance between the npAu surface and the attached sensitizer, but the chain length in axial position also contributes to the overall distance.

This is reflected by the obtained photocatalytic activities of the BsubPcs, where the derivative 8/H8 showed a higher increase in activity after immobilization compared to the sensitizers with a shorter and longer alkyl chain. This can be explained by the fact that this distance shows the best case for successful energy transfer from the npAu to the photosensitizer, whereas in the case of the undecynoxy-substituent the energy transfer efficiency decreases due to the large distance of around 3 nm. On the other hand, in the case of the propynoxy-substituted BsubPc photosensitizer with a distance of around 1 nm, electron transfer from the photosensitizer to the npAu support is dominant resulting in quenching of the excited state of the photosensitizer. In addition, BsubPcs are known to act as strong electron donors resulting in photoinduced charge transfer to the npAu enabling the needed energy transfer to molecular oxygen and thus resulting in smaller singlet sensitization activity.^35–37^

### Comparison of the different hybrid systems

**Fig. 8:**
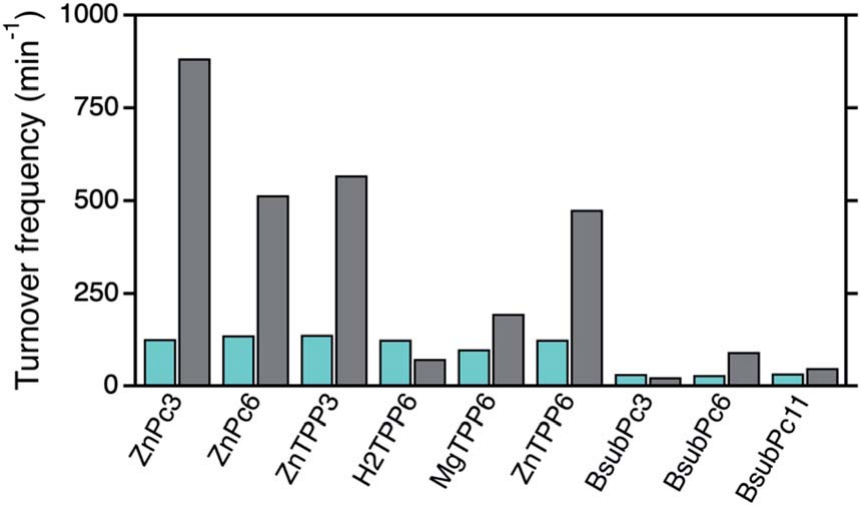
Comparison of the determined photocatalytic activities given as TOF values for DPF oxidation for the different photosensitizers when provided in solution (cyan) and after immobilization onto the npAu support (black).

For the final comparison of the photocatalytic activities of the different photosensitizers in solution as well as after immobilization onto the npAu surface, the corresponding TOF values for all systems were determined (Fig. S1–S9†). In the case of the ZnPc (1,2) and ZnTPP (3,6) photosensitizers, TOF values around 130 min^−1^ were observed for both derivatives when subjected to DPF oxidation providing the photosensitizer in solution (Fig. 8).

After immobilization onto the npAu, those photosensitizers all showed an increase in ^1^O_2_ sensitization activity, in both cases with a larger increase for the propynoxy-substituted ZnPc and ZnTPP derivatives (H1,H3). The origin of the difference in enhancement was shown to result from stronger axial coordination of the central metal ion to free azide groups from the SAM for the derivatives bearing shorter peripheral substituents.^25^ In addition, a larger enhancement of ^1^O_2_ sensitization was observed for the ZnPc derivatives compared to the ZnTPP photosensitizers due to a larger spectral overlap integral between the sensitizer absorption and the plasmon resonance of the npAu support.

The importance of the axial coordination of the central metal ion and its influence onto the overall activity of the hybrid material was shown exemplarily with the ZnTPP derivative (6/H6), where for comparison also the corresponding MgTPP (5/H5) and H_2_TPP (4/H4) derivatives were investigated. In the case of the MgTPP derivative, a weaker increase in activity was found after immobilization onto the npAu in comparison to the parent ZnTPP derivative, due to a weaker axial coordination of the magnesium central ion. The H_2_TPP derivative, where no axial coordination can be formed, was found to be the only derivative where no increase in activity after immobilization was observed as result of the lack of the axial coordination.

In contrast to the ZnPc and ZnTPP photosensitizers, the TOF values for DPF oxidation of the BsubPc derivatives in solution were found to be only around 30 min^−1^ as result of the molar extinction coefficients that are one order of magnitude smaller than for the other two classes of photosensitizers. After immobilization onto the npAu support, the BsubPc derivatives showed different properties compared to the ZnPc and ZnTPP derivatives, with only an increase in activity for the hybrid H8.

## Conclusions

In this study, different singlet oxygen photosensitizers were immobilized successfully onto a npAu support and studied in the photooxidation of DPF. Besides the previously studied hybrid systems bearing ZnPc derivatives as immobilized chromophores, hybrid systems comprising of different TPP and BsubPc derivatives were studied and compared to the parent hybrid system. The original ZnPc based hybrid systems showed the highest overall photocatalytic activity as well as the highest increase in activity after immobilization onto the npAu support.

In addition, most of the photosensitizers used in this study showed increased photocatalytic activity after immobilization, as result of synergistic energy transfer from the npAu plasmon resonance to the attached macromolecules, with different enhancement factors depending strongly on the position of the sensitizer absorption and the spectral overlap with the plasmon resonance. Therefore, the appropriate selection of the sensitizer can be based on the process conditions, offering a complete set of heterogeneous photocatalysts with absorption variability and wide applicability. In addition, the presented set of hybrid materials can be employed in further studies as building blocks towards highly active, panchromatic heterogeneous photocatalysts.

## Experimental

### Materials

Ag/Au alloy disks (70 : 30 at%, 5 mm diameter, 200 μm thickness) for npAu preparation were obtained according to a literature procedure.^18^ 6-Azidohexyl-1-thioacetate^38–40^ as building block for SAM formation, tris(benzyl-triazolylmethyl)amine (TBTA)^41,42^ as co-catalyst for the CuAAC reaction and the photosensitizers 2,9,16,23-tetrakis(2-propyn-1-yloxy)phthalocyanine zinc(ii) (ZnPc-3, 1),^43^ 2,9,16,23-tetrakis(5-hexyn-1-yloxy)phthalocyanine zinc(ii) (ZnPc-6, 2),^9^ and 5,10,15,20-tetrakis(4-(prop-2-yn-1-yloxy)phenyl)porphyrin zinc(ii) (ZnTPP-3, 3)^44^ were all synthesized as described elsewhere. The following chemicals and solvents were bought as indicated and were all used as received without further purification: Cu(MeCN)_4_PF_6_ (97%, Aldrich), hydroquinone (Merck), 2,5-diphenylfuran (DPF, >98.0%, TCI), EtOH (abs., reagent grade, VWR), THF (reagent grade, ≥99.0%, VWR), DMF (analytical reagent grade, ≥99.5%, VWR), HNO_3_ (analytical reagent grade, 65%, VWR), HNO_3_ (NORMATOM, ultra-pure for trace analysis, 67%, VWR) and HCl (NORMATOM, ultra-pure for trace analysis, 34%, VWR).

#### Synthesis of 5,10,15,20-tetrakis([hex-5-yn-1-yloxy]phenyl) porphyrin (H_2_TPP-6, 4)

Adapting a literature procedure,^45^ 5,10,15,20-tetra-(4-hydroxyphenyl) porphyrin (100 mg, 0.148 mmol) was dissolved in dry acetonitrile (50 mL) under nitrogen atmosphere. Subsequently, dry K_2_CO_3_ (205 mg, 1.48 mmol, dried at 180 °C under vacuum for 24 h) was added and the mixture was stirred at room temperature for one hour before 6-bromohex-1-yne (113 mg, 0.7 mmol) was added and the mixture was stirred under reflux for further 24 h. After the mixture was poured onto cold HCl (1 N), the blue-purple solid was isolated by filtration and redissolved in CH_2_Cl_2_ after drying. Then the organic layer was washed with aqueous Na_2_CO_3_ (250 mL, 5%) and H_2_O for three times. The organic layer was isolated, dried over Na_2_SO_4_ and the solvent was removed under reduced pressure to yield the desired compound as blue-purple crystals (87% yield). ^1^H NMR (600 MHz, CDCl_3_, ppm): *δ* = 8.89 (s, 8H), 8.13 (d, 8H), 7.30 (d, 8H), 3.95 (t, 8H), 2.3–2.2 (m, 8H), 1.92–1.88 (m, 4H), 1.77–1.73 (m, 8H), 1.56–1.48 (m, 8H), −2.68 (s, 2H). HR-MS: *m*/*z* = 999.48411 [M + H]^+^, calc. for C_68_H_63_N_4_O_4_^+^*m*/*z* = 999.48438. UV-Vis (CH_2_Cl_2_): *λ*_max_ [nm] = 421, 556, 594, 648.

### General procedure for the metalation of H_2_TPP-6

After a modified literature procedure,^46^ a solution of Mg(OAc)_2_ or Zn(OAc)_2_ (0.655 mmol) in MeOH was added to a solution of H_2_TPP (4, 0.488 mmol) in CHCl_3_ (30 mL) and the resulting mixture was stirred at room temperature for 1 h. After TLC confirmed completion of the metalation reaction, the mixture was poured onto H_2_O (100 mL) followed by extraction with CH_2_Cl_2_. The organic layer was separated, washed with H_2_O and brine and was dried over anhydrous Na_2_SO_4_. After filtration, the solvent was removed under reduced pressure to get the desired compound as a purple powder.

#### 5,10,15,20-Tetrakis(4-(hex-5-yn-1-yloxy)phenyl)porphyrin magnesium(ii) (MgTPP-6, 5)

Yield: 89%. ^1^H NMR (600 MHz, CDCl_3_, ppm): *δ* = 8.85 (s, 8H), 8.20 (m, 8H), 7.72 (m, 8H), 3.98 (t, 8H), 2.1–2.2 (m, 8H), 1.9–1.88 (m, 4H), 1.74–1.68 (m, 8H), 1.61–1.48 (m, 8H). HR-MS: *m*/*z* = 1051.50052 [M + H]^+^, calc. for C_70_H_67_MgN_4_O_4_^+^*m*/*z* = 1051.50073. UV-Vis (CH_2_Cl_2_): *λ*_max_ [nm] = 428, 566, 608.

#### 5,10,15,20-Tetrakis(4-(hex-5-yn-1-yloxy)phenyl)porphyrin zinc(ii) (ZnTPP-6, 6)

Yield: 92%. ^1^H NMR (600 MHz, CDCl_3_, ppm): *δ* = 8.88 (s, 8H), 8.21 (d, 8H), 7.77 (m, 12H), 3.98–3.94 (m, 8H), 2.22–2.18 (m, 8H), 1.92–1.88 (m, 4H), 1.74–1.70 (m, 8H), 1.57–1.52 (m, 8H). HR-MS: *m*/*z* = 1061.39767 [M + H]^+^, calc. for C_68_H_61_N_4_O_4_Zn^+^*m*/*z* = 1061.39788. UV-Vis (CH_2_Cl_2_): *λ*_max_ [nm] = 425, 558, 599.

### General procedure for BsubPc preparation

Based on a general literature procedure,^47^ boron subphthalocyanine chloride (100 mg, 0.231 mmol) and AgOTf (75 mg, 0.292 mmol) were dissolved in dry toluene (5 mL) and the mixture was stirred for 2 h under an argon atmosphere. After TLC showed full consumption of BSubPcCl, DIPEA (50 μL, 0.29 mmol) was added followed by addition of the appropriate *ω*-alkyn-1-ol (1.39 mmol) at which point a color change from purple to red was observed. After continued stirring for 20 h, the reaction mixture was passed through a short plug of SiO_2_ and eluted with EtOAc (100 mL). The filtrate was concentrated under reduced pressure, and the solid residue was dissolved in a small volume of CH_2_Cl_2_ before subjected to flash column chromatography (SiO_2_, gradient elution from 5–10% EtOAc/toluene) to afford the desired products with yields between 40–45% as pink powders.

#### B-(prop-2-ynyl-1-oxy)-subphthalocyaninato boron(iii) (BsubPc-3, 7)

^1^H NMR (600 MHz, CDCl_3_, ppm): *δ* = 8.76 (dd, 6H), 7.36 (dd, 6H), 1.55 (d, 2H), 1.31 (t, 1H). HR-MS: *m*/*z* = 450.13949 [M + H]^+^, calc. for C_27_H_16_BN_6_O^+^ (with ^11^B) *m*/*z* = 450.13965. UV-Vis (CH_2_Cl_2_): *λ*_max_ [nm] = 302, 516, 562.

#### B-(hex-5-ynyl-1-oxy)-subphthalocyaninato boron(iii) (BsubPc-6, 8)

^1^H NMR (600 MHz, CDCl_3_, ppm): *δ* = 8.74 (dd, 6H), 7.37 (dd, 6H), 1.61 (d, 2H), 1.32 (t, 1H), 1.22 (m, 2H), 1.12 (m, 4H). HR-MS: *m*/*z* = 493.19407 [M + H]^+^, calc. for C_30_H_22_BN_6_O^+^ (with ^11^B) *m*/*z* = 493.19427. UV-Vis (CH_2_Cl_2_): *λ*_max_ [nm] = 302, 514, 562.

#### B-(undec-11-ynyloxy)-subphthalocyaninato boron(iii) (BsubPc-11, 9)

^1^H NMR (600 MHz, CDCl_3_, ppm): *δ* = 8.8 (dd, 6H), 7.32 (dd, 6H), 1.59 (d, 2H), 1.34 (t, 1H), 1.17–1.05 (m, 16H) ppm. HR-MS: *m*/*z* = 563.27233 [M + H]^+^, calc. for C_35_H_32_BN_6_O^+^ (with ^11^B) *m*/*z* = 563.27252. UV-Vis (CH_2_Cl_2_): *λ*_max_ [nm] = 300, 516, 564.

### General procedure for the immobilization of the photosensitizers

npAu powder was prepared employing a free corrosion technique and functionalization with an azide-terminated SAM was performed employing an ethanolic solution of 6-azidohexyl-1-thioacetate as described previously by us.^10,25^ The immobilization of the photosensitizers was achieved by subjecting the as prepared azide-functionalized npAu powder to a solution of Cu(MeCN)_4_PF_6_ (931.8 μg, 2.5 μmmol), TBTA (1.326 mg, 2.5 μmol), hydroquinone (272.5 μg, 2.5 μmmol) and the respective photosensitizer (1–9, 0.15 μmol) dissolved in THF/H_2_O (5 mL, 3 : 1 v%). After a reaction time of 72 h, the reaction solution was discarded and the hybrid materials H1–H9 were repeatedly washed with THF to remove any unbound and physisorbed photosensitizer.

### Characterization methods

Characterization of the different synthesized photosensitizers was achieved by ^1^H NMR spectroscopy recorded on a 600 MHz Bruker AVANCE NEO spectrometer and HR-MS (ESI positive, direct injection) recorded with a Bruker Impact II spectrometer. All UV-Vis spectra were recorded using a UV-1600PC spectrometer from VWR with a resolution of 1 nm. Surface analysis of the npAu supports and the final hybrid materials was performed on a scanning electron microscope (SEM, Supra 40, Zeiss) operated at 15.0 kV acceleration voltage, 300 pA probe current and 10 mm working distance and equipped with a Bruker XFalsh 6/30 EDX detector. For the quantification of immobilized photosensitizer, the hybrid material (10 mg) was dissolved in ultrapure aqua regia (2 mL) prior to determination by ICP-MS using an iCAP-Q spectrometer form Thermo Fisher Scientific.

### DPF photooxidation over npAu hybrids

Photocatalytic oxidations were carried out in a self-built setup under irradiation with a 300 W Xe-arc lamp and a light intensity of 180 mW cm^−2^, as described elsewhere.^10,25^ In addition, a saturated aqueous mixture of NaNO_2_ and NaNO_3_ was used as filter solution for wavelength cutoff below 380 nm. For each photocatalytic experiment, the reaction vessel was filled with DMF (100 mL), the powder hybrid catalyst (25 mg) or the photocatalyst dissolved in DMF (100 μL, 0.1 nmol, molar ratio DPF/photosensitizer 30 000 : 1) was added and the setup was flushed with O_2_ for 10 min to achieve gas saturation of the solvent. Prior to irradiation, a DMF stock solution (500 μL) containing DPF (0.66 mg, 3 μmol) was added. The decrease of DPF concentration was followed *via* UV-Vis spectroscopy using Lambert Beers law at the absorption maximum at 327 nm (*ε*_327_ = 34 000 L mol^−1^ cm^−1^ in DMF). Photocatalytic activities were determined according to literature for immobilized sensitizers by determination of the turnover number (TON, [converted DPF] (mol)/[irradiated photosensitizer] (mol)) and turnover frequency (TOF, slope of the plot of TON *versus* reaction time (min^−1^)) for every hybrid system.^48^

## Conflicts of interest

There are no conflicts of interest to declare.

## Supplementary Material

RA-011-D1RA01331A-s001

## References

[cit1] Bayer P., Pérez-Ruiz R., Jacobi von Wangelin A. (2018). Stereoselective Photooxidations by the Schenck Ene Reaction. ChemPhotoChem.

[cit2] Hone D. C., Walker P. I., Evans-Gowing R., FitzGerald S., Beeby A., Chambrier I., Cook M. J., Russell D. A. (2002). Generation of Cytotoxic Singlet Oxygen *via* Phthalocyanine-Stabilized Gold Nanoparticles: A Potential Delivery Vehicle for Photodynamic Therapy. Langmuir.

[cit3] García Calavia P., Marín M. J., Chambrier I., Cook M. J., Russell D. A. (2018). Towards optimisation of surface enhanced photodynamic therapy of breast cancer cells using gold nanoparticle–photosensitiser conjugates. Photochem. Photobiol. Sci..

[cit4] Pibiri I., Buscemi S., Palumbo Piccionello A., Pace A. (2018). Photochemically Produced Singlet Oxygen: Applications and Perspectives. ChemPhotoChem.

[cit5] Spiller W., Kliesch H., Wöhrle D., Hackbarth S., Röder B., Schnurpfeil G. (1999). Singlet oxygen quantum yields of different photosensitizers in polar solvents and micellar solutions. J. Porphyrins Phthalocyanines.

[cit6] Shinohara H., Tsaryova O., Schnurpfeil G., Wöhrle D. (2006). Differently substituted phthalocyanines: Comparison of calculated energy levels, singlet oxygen quantum yields, photo-oxidative stabilities, photocatalytic and catalytic activities. J. Photochem. Photobiol., A.

[cit7] Gottschalk P., Paczkowski J., Neckers D. C. (1986). Factors influencing the quantum yields for rose bengal formation of singlet oxygen. J. Photochem..

[cit8] Sobbi A. K., Wöhrle D., Schlettwein D. (1993). Photochemical stability of various porphyrins in solution and as thin film electrodes. J. Chem. Soc., Perkin Trans. 2.

[cit9] Wichmann A., Schnurpfeil G., Backenköhler J., Kolke L., Azov V. A., Wöhrle D., Bäumer M., Wittstock A. (2014). A versatile synthetic strategy for nanoporous gold–organic hybrid materials for electrochemistry and photocatalysis. Tetrahedron.

[cit10] Steinebrunner D., Schnurpfeil G., Wichmann A., Wöhrle D., Wittstock A. (2019). Synergistic Effect in Zinc Phthalocyanine—Nanoporous Gold Hybrid Materials for Enhanced Photocatalytic Oxidations. Catalysts.

[cit11] Kotiaho A., Lahtinen R., Efimov A., Lehtivuori H., Tkachenko N. V., Kanerva T., Lemmetyinen H. (2010). Synthesis and time-resolved fluorescence study of porphyrin-functionalized gold nanoparticles. J. Photochem. Photobiol., A.

[cit12] Kotiaho A., Lahtinen R., Efimov A., Metsberg H.-K., Sariola E., Lehtivuori H., Tkachenko N. V., Lemmetyinen H. (2010). Photoinduced Charge and Energy Transfer in Phthalocyanine-Functionalized Gold Nanoparticles. J. Phys. Chem. C.

[cit13] Kotiaho A., Lahtinen R., Lemmetyinen H. (2011). Photoinduced processes in chromophore–gold nanoparticle assemblies. Pure Appl. Chem..

[cit14] Masilela N., Antunes E., Nyokong T. (2013). Axial coordination of zinc and silicon phthalocyanines to silver and gold nanoparticles: an investigation of their photophysicochemical and antimicrobial behavior. J. Porphyrins Phthalocyanines.

[cit15] Moeno S., Antunes E., Nyokong T. (2011). Synthesis and photophysical properties of a novel zinc photosensitizer and its gold nanoparticle conjugate. J. Photochem. Photobiol., A.

[cit16] Nombona N., Antunes E., Litwinski C., Nyokong T. (2011). Synthesis and photophysical studies of phthalocyanine–gold nanoparticle conjugates. Dalton Trans..

[cit17] Wittstock A. , Biener J., Erlebacher J. and Bäumer M., Nanoporous Gold: From an Ancient Technology to a High-Tech Material, RSC Publishing, RSC Nanoscience and Nanotechnology, 2012

[cit18] Wittstock A., Wichmann A., Bäumer M. (2012). Nanoporous Gold as a Platform for a Building Block Catalyst. ACS Catal..

[cit19] Wittstock A., Wichmann A., Biener J., Bäumer M. (2011). Nanoporous gold: a new gold catalyst with tunable properties. Faraday Discuss..

[cit20] Wittstock A., Bäumer M. (2014). Catalysis by Unsupported Skeletal Gold Catalysts. Acc. Chem. Res..

[cit21] Erlebacher J., Aziz M. J., Karma A., Dimitrov N., Sieradzki K. (2001). Evolution of nanoporosity in dealloying. Nature.

[cit22] Ding Y., Kim Y.-J., Erlebacher J. (2004). Nanoporous Gold Leaf: “Ancient Technology”/Advanced Material. Adv. Mater..

[cit23] Steinebrunner D., Schnurpfeil G., Wöhrle D., Wittstock A. (2020). Photocatalytic coatings based on a zinc(II) phthalocyanine derivative immobilized on nanoporous gold leafs with various pore sizes. RSC Adv..

[cit24] Detsi E., Salverda M., Onck P. R., De Hosson J. T. M. (2014). On the localized surface plasmon resonance modes in nanoporous gold films. J. Appl. Phys..

[cit25] Steinebrunner D., Schnurpfeil G., Kohröde M., Epp A., Klangnog K., Tapia Burgos J. A., Wichmann A., Wöhrle D., Wittstock A. (2020). Impact of photosensitizer orientation on the distance dependent photocatalytic activity in zinc phthalocyanine–nanoporous gold hybrid systems. RSC Adv..

[cit26] Abraham R. J., Leighton P., Sanders J. K. M. (1985). Coordination chemistry and geometries of some 4,4'-bipyridyl-capped porphyrins. Proton- and ligand-induced switching of conformations. J. Am. Chem. Soc..

[cit27] Satake A., Kobuke Y. (2005). Dynamic supramolecular porphyrin systems. Tetrahedron.

[cit28] Kobuke Y. (2006). Artificial Light-Harvesting Systems by Use of Metal Coordination. Eur. J. Inorg. Chem..

[cit29] Cremers J., Haver R., Rickhaus M., Gong J. Q., Favereau L., Peeks M. D., Claridge T. D. W., Herz L. M., Anderson H. L. (2018). Template-Directed Synthesis of a Conjugated Zinc Porphyrin Nanoball. J. Am. Chem. Soc..

[cit30] Rickhaus M., Jirasek M., Tejerina L., Gotfredsen H., Peeks M. D., Haver R., Jiang H.-W., Claridge T. D. W., Anderson H. L. (2020). Global aromaticity at the nanoscale. Nat. Chem..

[cit31] Bandi V., El-Khouly M. E., Nesterov V. N., Karr P. A., Fukuzumi S., D'Souza F. (2013). Self-Assembled *via* Metal–Ligand Coordination AzaBODIPY–Zinc Phthalocyanine and AzaBODIPY–Zinc Naphthalocyanine Conjugates: Synthesis, Structure, and Photoinduced Electron Transfer. J. Phys. Chem. C.

[cit32] D'Souza F., Smith P. M., Gadde S., McCarty A. L., Kullman M. J., Zandler M. E., Itou M., Araki Y., Ito O. (2004). Supramolecular Triads Formed by Axial Coordination of Fullerene to Covalently Linked Zinc Porphyrin−Ferrocene(s): Design, Syntheses, Electrochemistry, and Photochemistry. J. Phys. Chem. B.

[cit33] Marin D. M., Payerpaj S., Collier G. S., Ortiz A. L., Singh G., Jones M., Walter M. G. (2015). Efficient intersystem crossing using singly halogenated carbomethoxyphenyl porphyrins measured using delayed fluorescence, chemical quenching, and singlet oxygen emission. Phys. Chem. Chem. Phys..

[cit34] Dowds M., Nielsen M. B. (2021). Controlling the optical properties of boron subphthalocyanines and their analogues. Mol. Syst. Des. Eng..

[cit35] KC C. B., Lim G. N., D'Souza F. (2015). Charge Separation in Graphene-Decorated Multimodular Tris(pyrene)–Subphthalocyanine–Fullerene Donor–Acceptor Hybrids. Angew. Chem., Int. Ed..

[cit36] KC C. B., Lim G. N., D'Souza F. (2016). Effect of Spacer Connecting the Secondary Electron Donor Phenothiazine in Subphthalocyanine–Fullerene Conjugates in Promoting Electron Transfer Followed by Hole Shift Process. Chem.–Asian J..

[cit37] KC C. B., Lim G. N., Zandler M. E., D'Souza F. (2013). Synthesis and Photoinduced Electron Transfer Studies of a Tri(Phenothiazine)–Subphthalocyanine–Fullerene Pentad. Org. Lett..

[cit38] Ligeour C., Meyer A., Vasseur J.-J., Morvan F. (2012). Bis- and Tris-Alkyne Phosphoramidites for Multiple 5′-Labeling of Oligonucleotides by Click Chemistry. Eur. J. Org. Chem..

[cit39] Sodji Q. H., Patil V., Kornacki J. R., Mrksich M., Oyelere A. K. (2013). Synthesis and Structure–Activity Relationship of 3-Hydroxypyridine-2-thione-Based Histone Deacetylase Inhibitors. J. Med. Chem..

[cit40] Wu X., Ling C.-C., Bundle D. R. (2004). A New Homobifunctional p-Nitro Phenyl Ester Coupling Reagent for the Preparation of Neoglycoproteins. Org. Lett..

[cit41] Brassard C. J., Zhang X., Brewer C. R., Liu P., Clark R. J., Zhu L. (2016). Cu(II)-Catalyzed Oxidative Formation of 5,5′-Bistriazoles. J. Org. Chem..

[cit42] Chan T. R., Hilgraf R., Sharpless K. B., Fokin V. V. (2004). Polytriazoles as Copper(I)-Stabilizing Ligands in Catalysis. Org. Lett..

[cit43] Wöhrle D., Tsaryova O., Semioshkin A., Gabel D., Suvorova O. (2013). Synthesis and photochemical properties of phthalocyanine zinc(II) complexes containing o-carborane units. J. Organomet. Chem..

[cit44] Kushwaha D., Tiwari V. K. (2013). Click Chemistry Inspired Synthesis of Glycoporphyrin Dendrimers. J. Org. Chem..

[cit45] Molina-Mendoza A. J., Vaquero-Garzon L., Leret S., de Juan-Fernández L., Pérez E. M., Castellanos-Gomez A. (2016). Engineering the optoelectronic properties of MoS2 photodetectors through reversible noncovalent functionalization. Chem. Commun..

[cit46] He C., He Q., Deng C., Shi L., Zhu D., Fu Y., Cao H., Cheng J. (2010). Turn on fluorescence sensing of vapor phase electron donating amines *via* tetraphenylporphyrin or metallophenylporphrin doped polyfluorene. Chem. Commun..

[cit47] Gotfredsen H., Broløs L., Holmstrøm T., Sørensen J., Viñas Muñoz A., Kilde M. D., Skov A. B., Santella M., Hammerich O., Nielsen M. B. (2017). Acetylenic scaffolding with subphthalocyanines – synthetic scope and elucidation of electronic interactions in dimeric structures. Org. Biomol. Chem..

[cit48] Han X., Bourne R. A., Poliakoff M., George M. W. (2011). Immobilised photosensitisers for continuous flow reactions of singlet oxygen in supercritical carbon dioxide. Chem. Sci..

