# Supplementary material for: Comparison of the photocatalytic activity of novel hybrid photocatalysts based on phthalocyanines, subphthalocyanines and porphyrins immobilized onto nanoporous gold
Source: RSC Adv. 2021 Mar 18;11(19):11364–72. doi: 10.1039/d1ra01331a (PMC8695993; doi:10.1039/d1ra01331a)
Supplement: RA-011-D1RA01331A-s001 [file RA-011-D1RA01331A-s001.pdf]

Electronic Supplementary Material (ESI) for RSC Advances.
This journal is © The Royal Society of Chemistry 2021

Supporting Information

Comparison of the photocatalytic activity of novel hybrid photocatalysts based on phthalocyanines, subphthalocyanines and porphyrins immobilized onto nanoporous gold

David Steinebrunner,[a,b] Günter Schnurpfeil,[c] Jan Thayssen,[a,b,d] Jorge Adrian Tapia Burgos,[a,b] Andre Wichmann,[a] Dieter Wöhrle,*[c] and Arne Wittstock*[a,b]

[a] *Institute of Applied and Physical Chemistry and Center for Environmental Research and Sustainable Technology, University Bremen, Leobener Str. UFT, 28359 Bremen, Germany.*

[b] *MAPEX Center for Materials and Processes, University Bremen, Bibliothekstr. 1, 28359 Bremen, Germany.*

[c] *Organic and Macromolecular Chemistry, University Bremen, Leobener Str. NW2, 28359 Bremen, Germany.*

[d] *Institute for Organic and Analytical Chemistry, University Bremen, Leobener Str. UFT, 28359 Bremen, Germany.*

ESI-1 Determination of immobilized photosensitizer on npAu:

For the determination of the quantity of immobilized photosensitizer, 10 mg of the as prepared hybrid material was dissolved in ultra pure *aqua regia* (2 mL) and subjected to ICP-MS measurements. The quantities of the immobilized photosensitzers were then calculated from the concentrations of the central ion (Zn or B, Table S1) determined by ICP-MS. From the obtained amounts, the fractions of irradiated photosensitizer were calculated according to a literature procedure based on the penetration depth of visible light into the npAu material of 300 nm (Table S1).[1]

The amounts of irradiated photosensitzer were used for further quantification of the photocatalytic activites by calculation of the corresponding turnover numbers (TON, [converted DPF] (mol) / [irradiated photosensitizer] (mol)) and turnover frequencies (TOF, slope of the plot TON vs. reaction time (min^{-1})).

Table. S1: Overview over the different hybrid systems prepared by immobilization of the photosensitizer onto npAu. Hybrid – abbreviation used in the text for the corresponding hybrid system, $Sensitizer_{npAu}$ – immobilized sensitizer content given as µg/g hybrid catalyst as determined by ICP-MS measurements, and $Sensitizer_{Irr.}$ – determined photosensitizer amount immobilized on npAu and irradiated during the photocatalytic measurements.

**Hybrid** (Sensitizer on npAu):	**H1** (ZnPc-3)	**H2** (ZnPc-6)	**H3** (ZnTPP-3)	**H6** (ZnTPP-6)	**H7** (BsubPc-3)	**H8** (BsubPc-6)	**H9** (BsubPc-11)
$Sensitizer_{npAu}$ [µg/g]:	165.9	156.7	100.7	117.6	601.1	406.2	394.3
$Sensitizer_{Irr.}$ [mol]:	1.5 x 10^{-10}	1.4 x 10^{-10}	1.0 x 10^{-10}	1.1 x 10^{-10}	5.5 x 10^{-10}	3.7 x 10^{-10}	3.6 x 10^{-10}

ESI-2 Supplementary data for the photooxidation of DPF with ZnPc-3

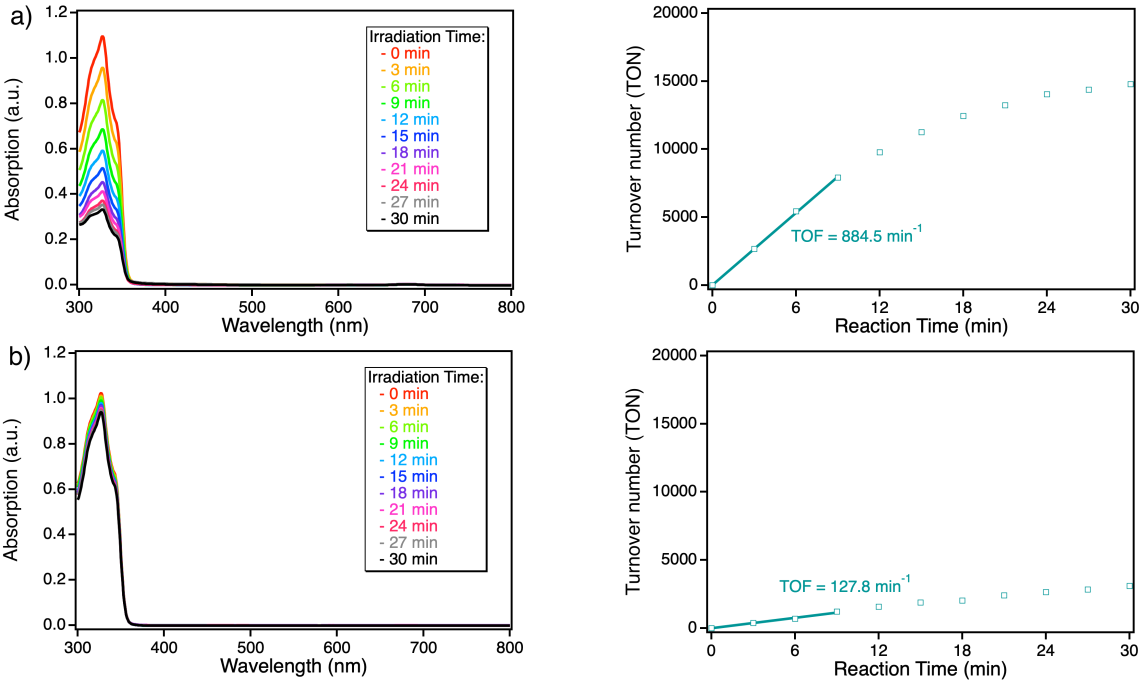

Fig. S1: UV-Vis spectra for the photooxidation of DPF with (a) **H1** as hybrid photocatalyst or (b) the photosensitizer ZnPc-3 (**1**, 1x10^{-10} mol) in solution.

ESI-3 Supplementary data for the photooxidation of DPF with ZnPc-6

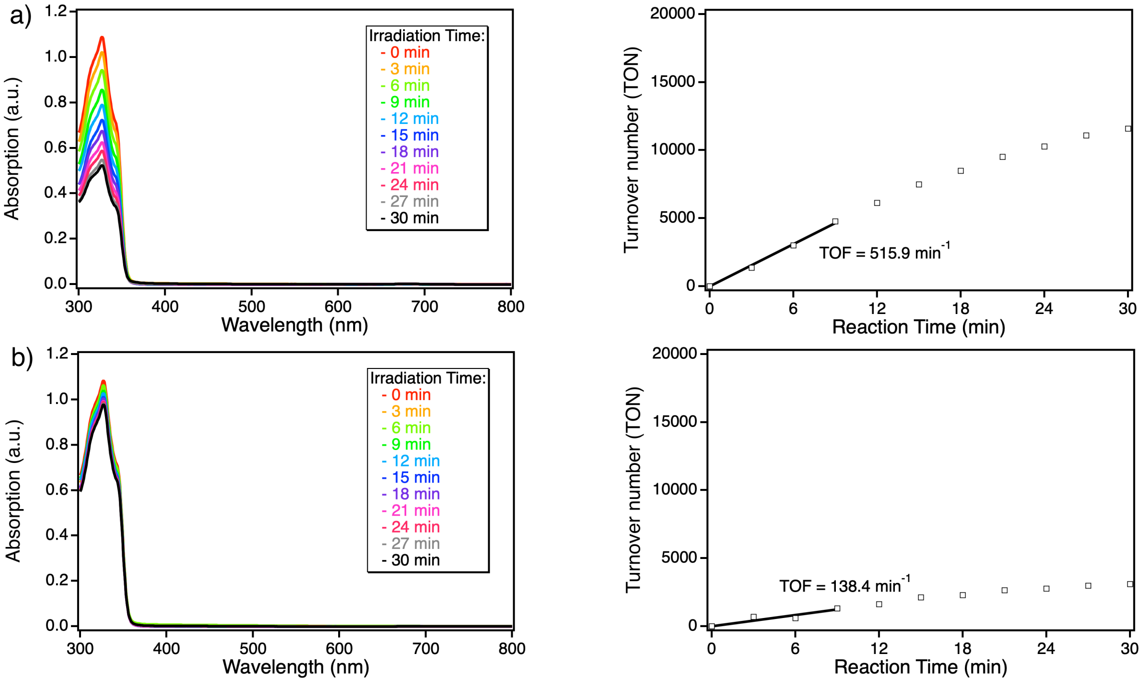

Fig. S2: UV-Vis spectra for the photooxidation of DPF with (a) **H2** as hybrid photocatalyst or (b) the photosensitizer ZnPc-6 (**2**, 1x10^{-10} mol) in solution.

ESI-4 Supplementary data for the photooxidation of DPF with ZnTPP-3

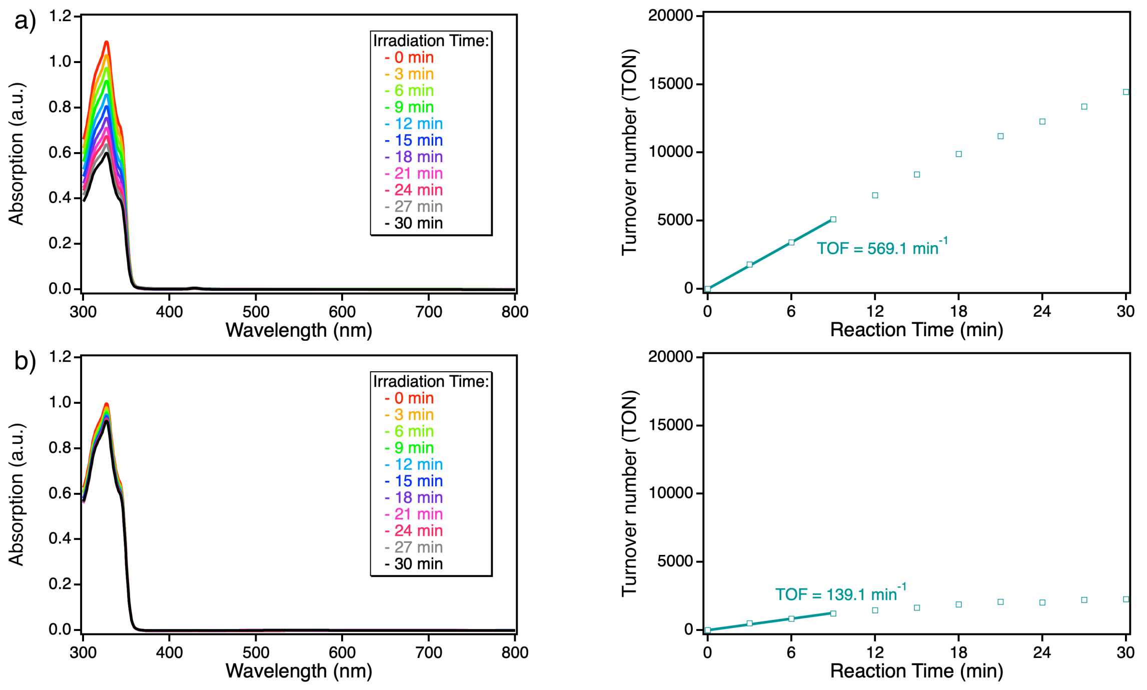

Fig. S3: UV-Vis spectra for the photooxidation of DPF with (a) **H3** as hybrid photocatalyst or (b) the photosensitizer ZnTPP-3 (**3**, $1x10^{-10}$ mol) in solution.

ESI-5 Supplementary data for the photooxidation of DPF with H_2TPP-6

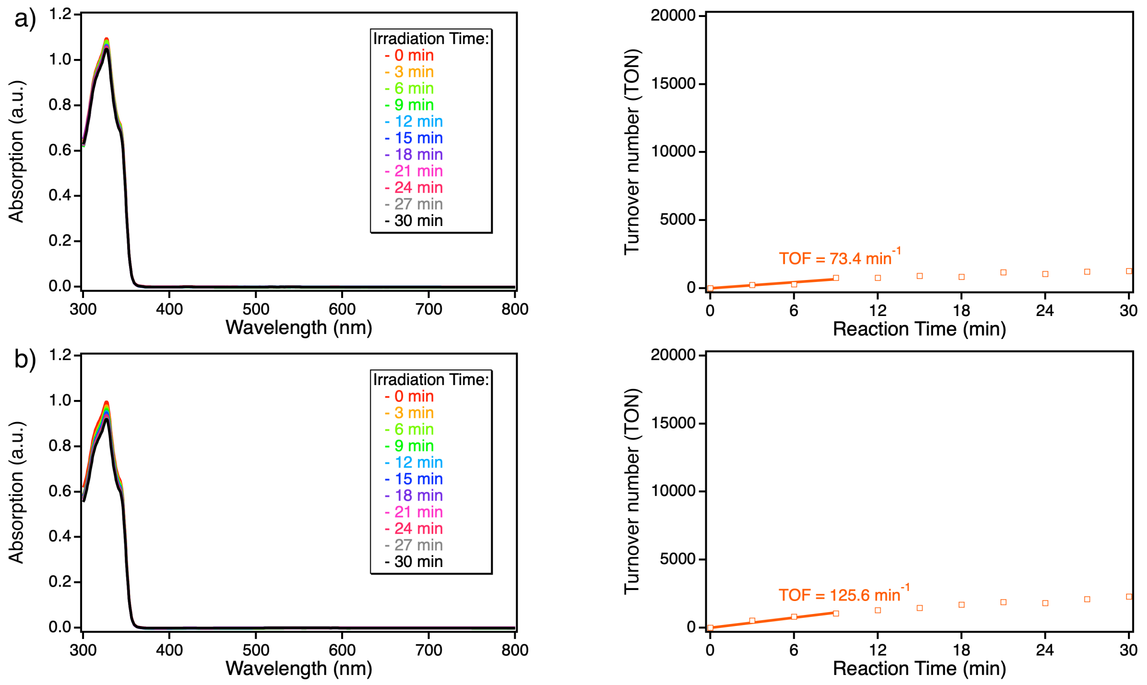

Fig. S4: UV-Vis spectra for the photooxidation of DPF with (a) **H4** as hybrid photocatalyst or (b) the photosensitizer H_2TPP-6 (**4**, $1x10^{-10}$ mol) in solution.

ESI-6 Supplementary data for the photooxidation of DPF with MgTPP-6

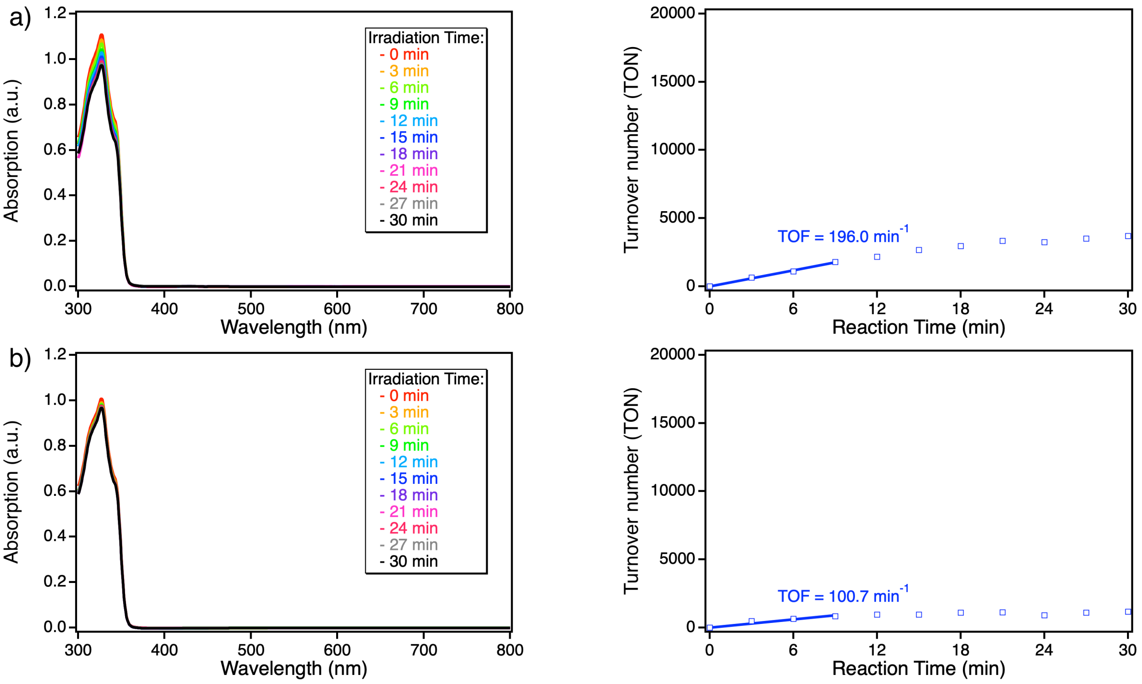

Fig. S5: UV-Vis spectra for the photooxidation of DPF with (a) **H5** as hybrid photocatalyst or (b) the photosensitizer MgTPP-6 (**5**, 1x10^{-10} mol) in solution.

ESI-7 Supplementary data for the photooxidation of DPF with ZnTPP-6

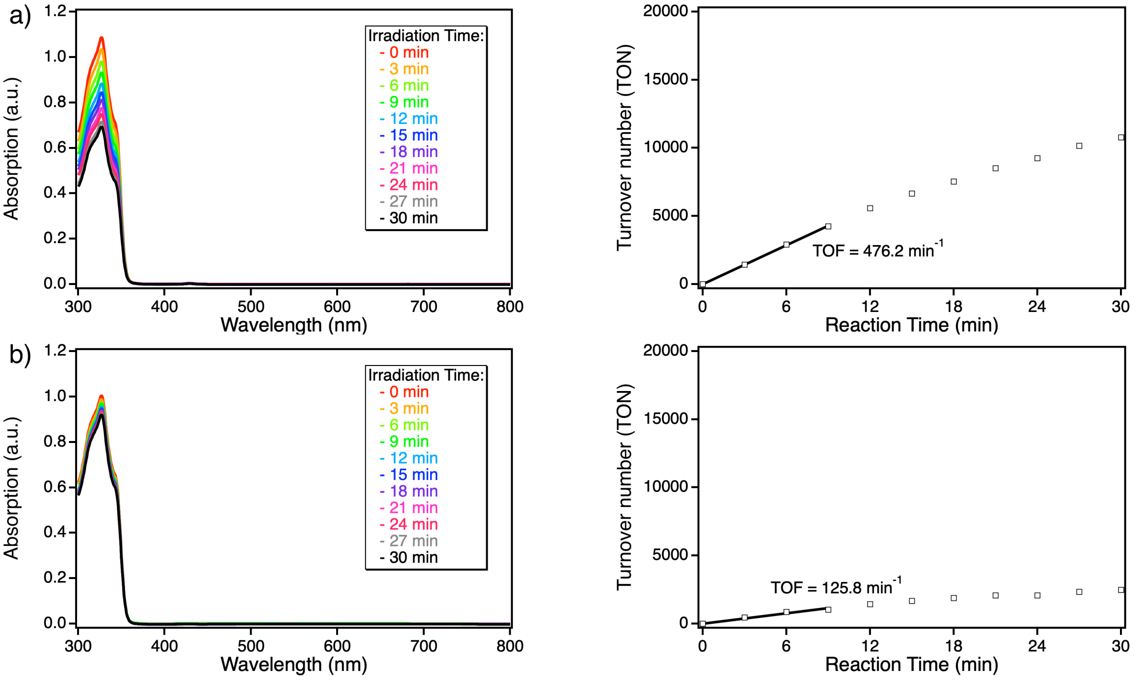

Fig. S6: UV-Vis spectra for the photooxidation of DPF with (a) **H6** as hybrid photocatalyst or (b) the photosensitizer ZnTPP-6 (**6**, 1x10^{-10} mol) in solution.

ESI-8 Supplementary data for the photooxidation of DPF with BsubPc-3

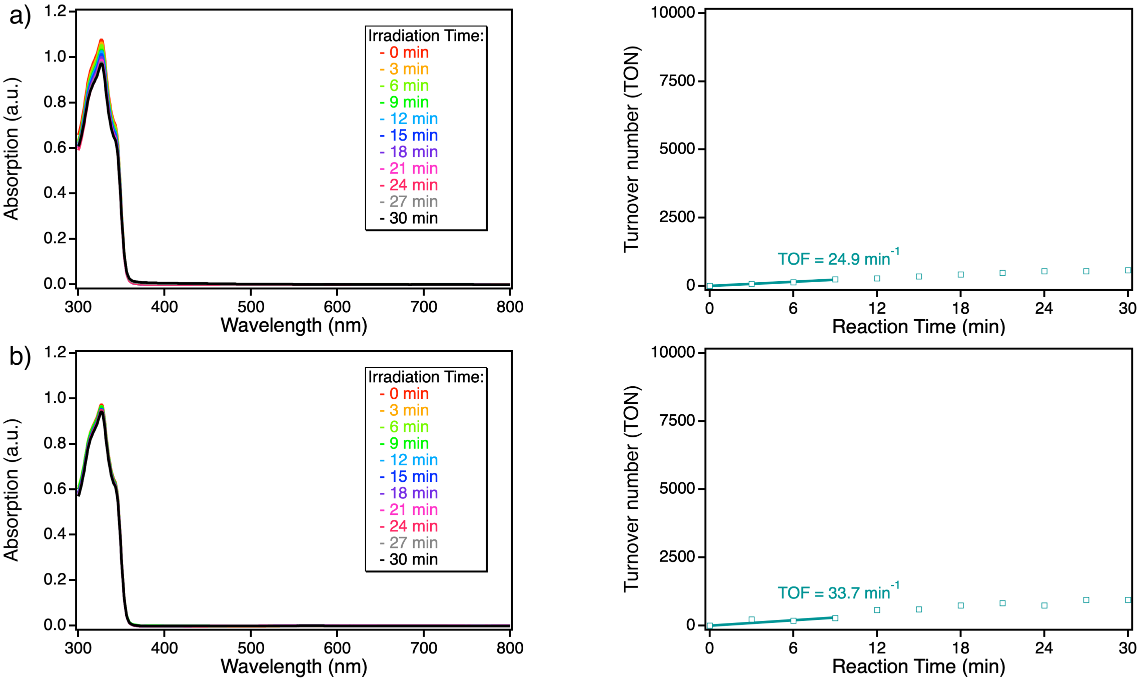

Fig. S7: UV-Vis spectra for the photooxidation of DPF with (a) **H7** as hybrid photocatalyst or (b) the photosensitizer BsubPc-3 (**7**, $1x10^{-10}$ mol) in solution.

ESI-9 Supplementary data for the photooxidation of DPF with BsubPc-6

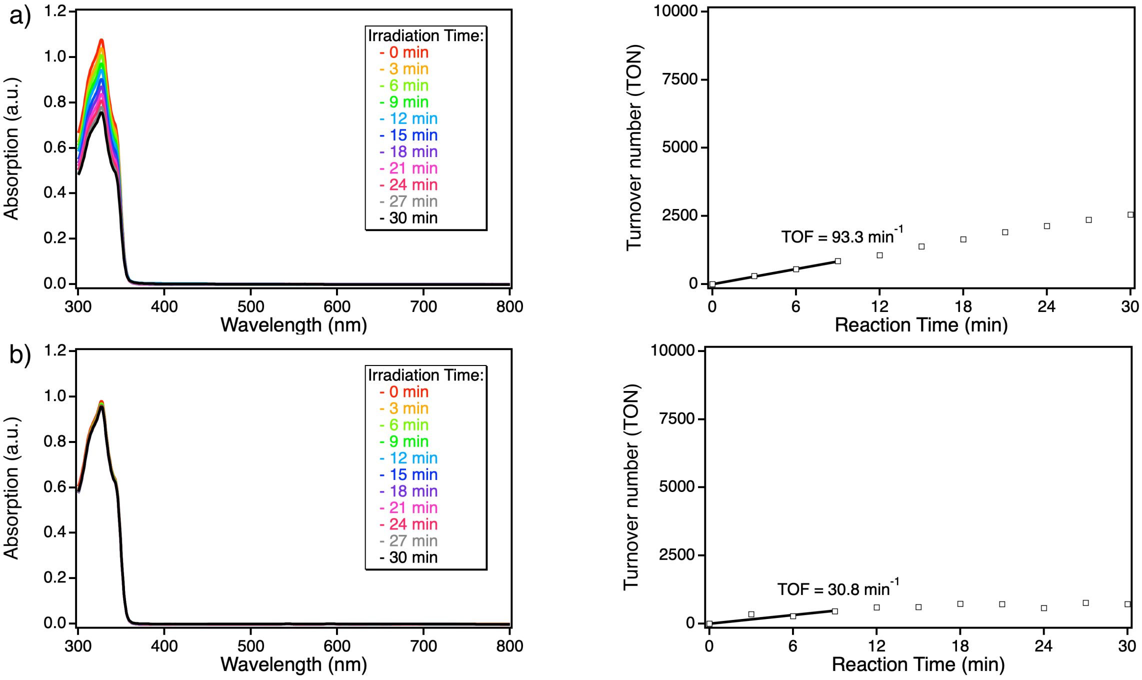

Fig. S8: UV-Vis spectra for the photooxidation of DPF with (a) **H8** as hybrid photocatalyst or (b) the photosensitizer BsubPc-6 (**8**, $1x10^{-10}$ mol) in solution.

ESI-10 Supplementary data for the photooxidation of DPF with BsubPc-11

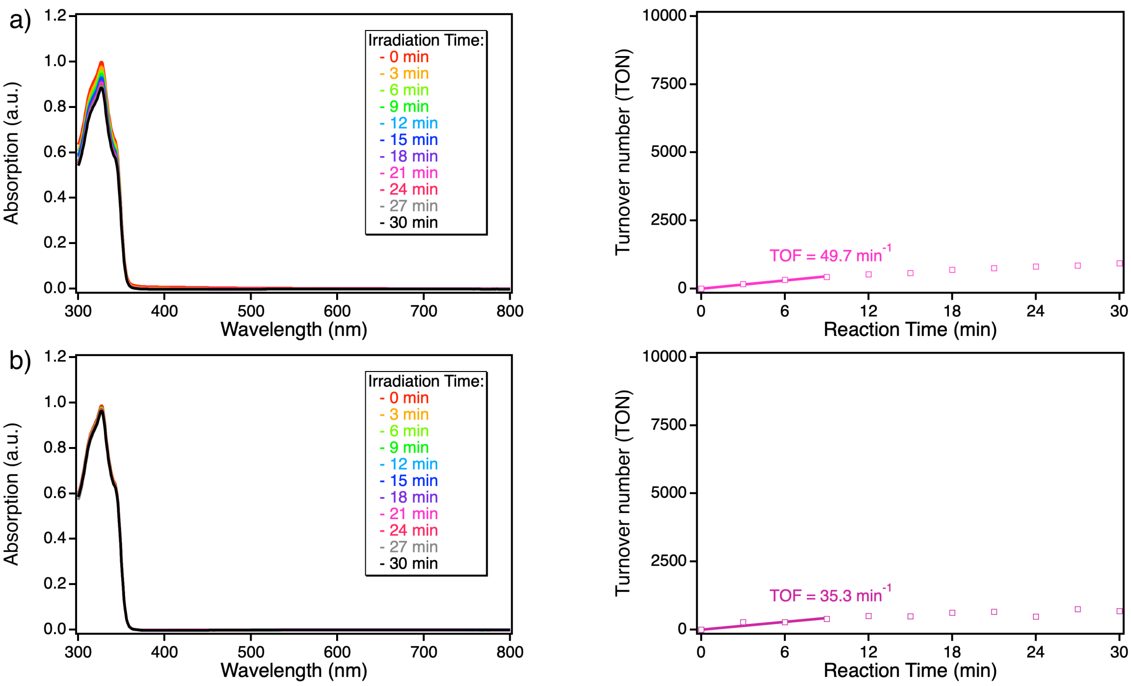

Fig. S9: UV-Vis spectra for the photooxidation of DPF with (a) **H9** as hybrid photocatalyst or (b) the photosensitizer BsubPc-11 (**9**, $1x10^{-10}$ mol) in solution.

References:

[1] D. Steinebrunner, G. Schnurpfeil, A. Wichmann, D. Wöhrle and A. Wittstock, Synergistic Effect in Zinc Phthalocyanine – Nanoporous Gold Hybrid Materials for Enhanced Photocatalytic Oxidations, *Catalysts*, 2019, **9**, 555.
